# Competency-based simulation assessment of resuscitation skills in emergency medicine postgraduate trainees – a Canadian multi-centred study

**Published:** 2016-03-31

**Authors:** J. Damon Dagnone, Andrew K. Hall, Stefanie Sebok-Syer, Don Klinger, Karen Woolfrey, Colleen Davison, John Ross, Gordon McNeil, Sean Moore

**Affiliations:** 1 Department of Emergency Medicine, Queen’s University; 2 Faculty of Education, Queen’s University; 3 Department of Emergency Medicine, Western University; 4 Department of Public Health Sciences, Queen’s University; 5 Department of Emergency Medicine, Dalhousie University; 6 Department of Emergency Medicine University of Calgary; 7 Department of Emergency Medicine, University of Ottawa

## Abstract

**Background:**

The use of high-fidelity simulation is emerging as a desirable method for competency-based assessment in postgraduate medical education. We aimed to demonstrate the feasibility and validity of a multi-centre simulation-based Objective Structured Clinical Examination (OSCE) of resuscitation competence with Canadian Emergency Medicine (EM) trainees.

**Method:**

EM postgraduate trainees (*n*=98) from five Canadian academic centres participated in a high fidelity, 3-station simulation-based OSCE. Expert panels of three emergency physicians evaluated trainee performances at each centre using the Queen’s Simulation Assessment Tool (QSAT). Intraclass correlation coefficients were used to measure the inter-rater reliability, and analysis of variance was used to measure the discriminatory validity of each scenario. A fully crossed generalizability study was also conducted for each examination centre.

**Results:**

Inter-rater reliability in four of the five centres was strong with a median absolute intraclass correlation coefficient (ICC) across centres and scenarios of 0.89 [0.65–0.97]. Discriminatory validity was also strong (*p* < 0.001 for scenarios 1 and 3; *p* < 0.05 for scenario 2). Generalizability studies found significant variations at two of the study centres.

**Conclusions:**

This study demonstrates the successful pilot administration of a multi-centre, 3-station simulation-based OSCE for the assessment of resuscitation competence in post-graduate Emergency Medicine trainees.

## Introduction

The assessment of resuscitation skills in postgraduate medical trainees is moving towards competency-based methods, and away from knowledge-based examination.^1^,^2^ As noted by Miller and colleagues,^3^ there is a progression from “knows” to “knows how,” “shows how” and “does” through medical training. Thus, it is necessary to assess not only technical knowledge, but also practical competencies such as clinical reasoning and teamwork. This move towards appropriate competency-based assessment in postgraduate education has been endorsed by Accreditation Council for Graduate Medical Education (ACGME, USA) and Royal College of Physicians and Surgeons of Canada (RCPSC), as well as at the 2010 Ottawa Conference “Assessment of Competence in Medicine and the Healthcare Professions.”^4^–^6^

High fidelity simulations using computer-controlled mannequins with mechanized movements, cues, and simulated vital signs are being used throughout medical education to emulate real patient encounters.^7^,^8^ This simulation-based medical education gives trainees opportunities to practice, while simultaneously allowing them opportunities to develop their teamwork and communication skills.^9^,^10^ A recent meta-analysis highlighted this method of training to be superior to opportunistic exposures in clinical medical education in achieving specific clinical skill acquisition goals.^11^ As a result, there has been a dramatic increase in the use of high-fidelity simulation in Emergency Medicine (EM) and Anesthesia.^6^ In fact, the ACGME has required simulation be directly incorporated into postgraduate EM curricula - not as a separate adjunct, but as the primary education strategy for topics that have been deemed best taught in a simulation format.^12^ Despite the recent development and integration of high-fidelity mannequin-based simulation in post-graduate medical education, there have been limited advancements in integrating simulation within competency-based assessment systems.^13^

An examination of the literature for simulation-based assessment within EM and across the other specialties reveals a deficit of easily modifiable and useful assessment tools for dynamic resuscitation skill performance. Many studies have been published within Anesthesia, Pediatrics, and EM that demonstrate excellent discriminatory ability and inter-rater reliability in the assessment of specific ACLS,^14^–^17^ CRM,^18^,^19^ or team competency based skills.^20^–^22^ However, these assessments lack the appropriate metrics for widespread use by postgraduate licensing bodies and training programs.^8^ This lack of metrics is further supported by a recent systematic review of technology-enhanced simulation in the assessment of health professionals that states evidence for validity of previous studies is sparse with “room for improvement.”^23^,^24^ Although many studies have repeatedly illustrated strong assessment tool performance for measuring defined outcomes, they have often been limited in scope, used simple checklists or a specified algorithmic approach, and have failed to satisfy the “unified model” for validity.^24^,^25^ As a result, there remains a great need for valid and reliable simulation-based assessment tools for assessing competency-based resuscitation skills of medical trainees.

Valid and reliable simulation-based competency assessment of resuscitation skills would be beneficial to postgraduate licensing bodies and training programs both in EM and in other specialties.^8^ In order for simulation-based activities to be used for assessment of resuscitation skills, appropriate metrics must be constructed. In Pediatrics and Anesthesia,^26^–^30^ the ability of assessment tools to discriminate between trainees of different training levels, using high-fidelity simulation case scenarios has been recently demonstrated with reasonable inter-rater reliability. As well, a simulation-based Objective Structured Clinical Examination (OSCE) has been incorporated into the Israeli National Board Examination in Anesthesia.^31^ This is the first case of this type of examination being used for such high-level certification.

The Queen’s Simulation Assessment Tool (QSAT) was developed to allow for competency-based assessment of any EM resident trainee in a simulated resuscitation under a number of different clinical and team-based circumstances.^32^ Building on previous validation work done at Queen’s University in competency-based simulation assessment,^32^,^33^ a simulation-based OSCE was administered at five different academic training centres across Canada to evaluate postgraduate trainees’ resuscitation skills using the QSAT. This study had three primary aims. Firstly, we wanted to determine if multiple, efficiently trained raters across five locations could provide reliable QSAT scores to post graduate medical trainees. Secondly, we hoped to determine whether or not the three resuscitation scenarios developed for and constituting the simulation-based OSCE could discriminate among postgraduate EM trainees based on their years of training. Lastly, we were interested in gathering evidence to support the number of stations or raters needed to provide reliable estimates of trainee competence in resuscitation skills.

## Methods

### Study design

A prospective observational design was employed to study a multi-centre, simulation-based OSCE for assessment of resuscitation skills in EM postgraduate trainees. A previously developed and validated simulation-based resuscitation assessment tool was used to assess the performances of all trainees.

#### Assessment system

The QSAT was designed to be simple and modifiable in assessing specific and generalized resuscitation parameters.^32^ It is unique in its basic framework in that it can be modified for specific clinical scenarios. The QSAT uses a standardized format with two components: 1) four domain scores (initial assessment, diagnostic approach, therapeutic approach, and communication skills, and 2) a single global assessment score (GAS). All domain scores and the GAS are based on a 5-point Likert rating scale (1=inferior to 5=superior) with descriptors for each numerical score. Each domain score also contains anchored skills to assist in scoring (sample assessment available from authors upon request).

### Study setting

The study took place over an 18-month period at five academic University simulation centres across Canada: 1) Queen’s University (Kingston Resuscitation Institute Lab), 2) University of Toronto (Sunnybrook campus), 3) University of Ottawa (Ottawa Civic Campus), 4) University of Calgary (Foothills Hospital Campus), and 5) Dalhousie University (Queen Elizabeth II Campus, Halifax). Multiple models of high-fidelity simulation mannequins were employed: Gaumard Hal^®^ and Susie^®^ (Gaumard Scientific, Miami, Fl), and Laerdal SimJunior^®^ (Laerdal Medical Canada, Ltd., Toronto, ON). All simulations were run by a simulation technician and a member of the research team (AKH, DD). Physiologic parameters (e.g. vital signs, eye opening, breath sounds) were adjusted using a predetermined set of palettes. The progression of palettes followed the therapeutic actions of the trainees during the OSCE scenario in a standardized fashion. The simulation lab at each centre was set-up on each occasion to re-create the physical environment of an emergency department resuscitation bay, and all necessary equipment or tools were available to the trainees.

### Study participants

A total of 98 postgraduate trainees, EM physician trainees from the Fellow of the Royal College of Physicians & Surgeons of Canada – Emergency Medicine (FRCPC-EM) program and the Certificate of the College of Family Physicians of Canada – Special Competence in Emergency Medicine (CCFP-EM) program participated in this study. Participation was voluntary and written consent was obtained. Approval was obtained from the Ethics Review Boards at all five participating centres.

### Scenarios and assessment tool development

Three previously developed and validated standardized emergency department resuscitation scenarios, each with a corresponding QSAT, were used.^32^ All scenarios were based on core content and objectives for EM postgraduate programs, as outlined by the RCPSC. An expert panel of EM faculty, with training in high fidelity simulation-based instruction, developed the scenarios. The chosen scenarios were: 1) Acute Congestive Heart Failure with Respiratory Distress, 2) Subarachnoid Hemorrhage with Decreased Level of Consciousness, and 3) Sympathomimetic Stimulant Ingestion. The scenario duration was 7 minutes for each station. Each scenario included scripted roles and clear instructions for the trained actors and the simulation technician.

### OSCE administration and evaluation

The 3-station, simulation-based OSCE was administered at each of the five academic centres. Each trainee completed the 3 standardized scenarios over a total of 30 minutes. Each trainee was provided with verbal instructions for the OSCE prior to their participation and given one minute to read a written scenario stem immediately prior to performing each scenario. The trainees’ performances were recorded from 3 fixed camera angles to allow adequate views of the trainee, the mannequin, and the cardiac monitors (Appendix 1). Members of the research team observed and directed each session from an obscured location. Each candidate received a formal debriefing by an associated faculty member immediately following their performance for the purposes of formative feedback. For each centre, the videotaped performances were stored on a secure laptop computer and subsequently rated by three independent, blinded content experts. All raters were EM faculty with 5 years or more experience and specific training in simulation-based education. To minimize bias, the raters were not faculty physicians at the same centre as the trainees they were assessing, and the raters were not given any information about the trainees’ identities or level of training. Each centre had a different group of 3 raters; the same 3 raters scored all of the trainees at a centre. In order to standardize evaluations, all raters underwent a 3-hour training session, led by an investigator, on the use of the assessment tools. They practiced using the assessment tool with standardized recorded performances designed specifically for orientation.

### Statistical analysis

Data analyses were performed using IBM SPSS version 22.0. The initial analyses focused on the inter-rater reliability in order to establish that the raters could provide consistent scores for the residents across centres. Intraclass correlation coefficients (ICCs) are a preferred method to determine the average rate of absolute agreement when there are two or more raters scoring the same trainees.^34^ Given that distinct groups of three raters assessed residents from different locations, separate two –way random ICCs were calculated to determine the average level of absolute rater agreement across the three raters at each centre for each scenario. The set of ICCs from the centres and items were compared to determine if the raters were able to use the QSAT consistently.

Our next set of analyses focused on the ability of the QSAT to discriminate across trainees based on their level of training. Trainees were separated based on their level of training (junior FRCP [postgraduate year (PGY) 1,2], CCFP-EM [PGY 3], and senior FRCP [PGY 3,4,5]). These groups were compared using three one-way (level of training) ANOVAs, using the three scenarios as dependent variables. For each scenario, the null hypothesis was that trainees’ scores would not differ based on their level of training. Discriminatory evidence was provided if trainees with higher levels of training obtained significantly higher scores on the 3 scenarios. An omnibus *F*-test was conducted to determine if any group differences exited. Given that there were three scenarios, a Bonferroni correction was used to reduce the likelihood of a Type I error. Thus the null hypothesis was rejected if *α* <0.017 (0.05/3). If a significant group difference was found, post hoc analyses of the main effects were conducted using the Tukey’s HSD method (*α* <0.017).

Finally, a Generalizability study (*G-*study) was conducted for trainees’ scores at each centre (Trainee X Rater X Scenario) to determine the variance components and *G-*coefficients. *G-*Studies are useful for initial test design research as they can be used to identify the sources of score variation and then to help determine the optimal number of stations needed for reliable estimates of trainee performance. *G-*studies identify variance components for main and interaction effects. For these analyses, trainees were the object of measurement and raters and scenarios served as the potential sources of error. The calculated variance components were subsequently used to conduct a series of Decision studies (*D-*studies) to determine the generalizability that could be obtained using different combinations of raters and scenarios, maximizing efficiency and accuracy of assessments.

## Results

A total of 103 EM postgraduate trainees (FRCP & CCFP-EM) from Queen’s University, Ottawa University, the University of Toronto, the University of Calgary, and Dalhousie University participated in the study. Each centre had unequal distributions of trainee level participation, both within centre and across centres. Three blinded expert EM examiners (raters) from each centre rated all trainee from one of the other four centres. This was done in all 5 centres. On the rare occasion when there were technical errors with the recorded performances (sound, video) or if expert data sheets revealed aberrancies (multiple scores circled or incomplete entries), a participant’s data were excluded. As a result, the final sample consisted of 98 trainees with complete data for all 3 scenarios (Table 1).

The intraclass correlation coefficients (ICC), which provide a measure of the inter-rater reliability of the ratings, of the QSAT ratings for each scenario are shown in Table 2. The average (3 rater) absolute ICC of the QSAT scores for each scenario were high to very high (0.84–0.97) across four of the five study centres, with moderate to moderately high values for one of the centres (0.64–0.80).^35^ The median ICC across scenarios and centres, a superior measure of central tendency for reliabilities and correlations, was 0.89. Overall, these ICC results indicated the centre-level data provided reliable measures of trainee performance. Given the consistency of ratings within each site, the scenario scores from each of the centres were combined for the purposes of examining the discriminatory capability of the QSAT (there was no intention to compare centres in terms of trainees’ scores).

A series of three one-way ANOVAs were used to compare trainees based on their level of training for each of the three scenarios. Significant between group differences were found for each of the scenarios (Scenario 1, *F*_2,96_ = 12.12, *p* < .001; Scenario 2, *F*_2,96_ = 9.82, *p* < .001; Scenario 3, *F*_2,96_ = 11.49, *p* < .001). *Post hoc* analyses indicated that Jr-FRCP (PGY-1,2) trainees scored significantly lower than Sr-FRCP trainees (PGY-3,4,5) (Table 3). No significant differences were found between CCFP-EM (PGY-3) trainees when compared to the Jr- and Sr- FRCP trainees.

Finally, a Generalizability study (*G*-study) was conducted using a fully crossed trainee by rater by scenario (T × R × S) design. Since each group of raters only marked the trainees from a single centre, separate analyses were conducted for each of the 5 centres. The estimated variance components, the relative contributions to score variance, and the *G*-coefficients are provided in Table 4. With one exception, the largest source of variance was the trainee by scenario interaction. This means that trainees’ performances varied from scenario to scenario. This finding is not surprising as the trainee by scenario interaction in *G-*theory is essentially highlighting content specificity, which has been previously documented in the medical education literature.^36^

*D*-studies were conducted to evaluate the effectiveness of alternative designs with differing numbers of facets for each of the administered examinations (Table 4). Coefficients had significant variability based on centre, with Queen’s, Ottawa, and Dalhousie having the most similar estimates. The *D*-studies suggest that increasing the number of scenarios per OSCE to between 6 and 9 with only a single rater per station would produce *G*-coefficients ranging from 0.81 to 0.91. For instance, with 6 scenarios and 1 rater, the range of *G*-coefficients would be 0.83–0.91. As noted previously, the subsamples from specific centres resulted in differences in the centre level estimates. In contrast, the Toronto and Calgary centres demonstrated minimal improvements in *G*-coefficients (0.31–0.76) when increasing the number of scenarios regardless of the number of raters.

## Discussion

Simulation-based education has become an integral part of postgraduate training of resuscitation skills for EM, Anesthesiology, Internal Medicine, Surgery, and Critical Care.^7^ Understandably, there has been a broad call for the development and implementation of tools to assess competency of postgraduate medical trainees.^4^–^6^ Within EM, validated competency-based assessment tools which demonstrate validity and reliability sufficient to be used for learning progress, readiness for practice, and high stakes decision making, have been recommended by numerous accreditation bodies.^5^,^28^,^29^,^37^ This pilot study examined the performance of a previously validated simulation-based assessment tool (the QSAT) in a 3-station resuscitation OSCE at 5 post-graduate EM training programs across Canada.^32^ Despite the challenges of standardizing the environmental testing conditions for all trainees, recruiting trainees for participation, and orienting new expert examiners at each of the five separate centres, the QSAT performed well, demonstrating its promise for high stakes or summative assessment contexts.

The inter-rater reliability (ICCs) of the QSAT was high to very high (0.84–0.97) in all but one centre (Toronto). The lower values at the Toronto centre were likely due to the raters of the Toronto trainees noticing various aspects of poor performance, resulting from a restricted, homogenous sample.^38^ Overall, the set of findings demonstrate that our study methodology was successful in training experts to be reliable raters.

Importantly, the QSAT was found to be able to differentiate between levels of trainees, highlighting its effectiveness to discriminate between likely levels of competence. Residents with higher levels of training (SR-FRCP) consistently demonstrated higher scores compared to those with lower levels of training (JR-FRCP). This means the 3-stations were able to differentiate between varying levels of trainee competence and could be used in future high stakes summative exams to assess thresholds of performance. It is important to note that a third group of trainees was also examined: the CCFP-EM senior resident group. This group was specifically separated out from the Jr-FRCP and Sr-FRCP groups for analysis because of their different certification model. The CCFP-EM group, with two years of Family Medicine training followed by a single year of EM, is a very heterogeneous group with high variability in their expected performances for resuscitation scenarios. There were no significant differences between CCFP-EM trainees when compared to either the Jr-FRCP or Sr-FRCP groups. Admittedly, the smaller CCFP-EM sample likely impacted the analyses. Our future studies will revisit this CCFP-EM trainee group while also continue to examine the different levels within the FRCP.

Lastly, the generalizability of the QSAT was examined, with the intention of providing guidance for the subsequent use of QSAT in actual testing conditions. In our study, the largest sources of variance were the trainees, followed by the trainee × scenario component at four of the five centres.^39^ Across all five centres, there was minimal variance for the rater and scenario components individually. These findings indicate that more than three cases would be required to increase the generalizability to a sufficient level for high-stakes assessments of trainees. The *D*-study results which found that an ideal number of scenarios to achieve a *G*-coefficient greater than 0.8 with only 1 rater would be greater than 6, which is consistent with existing literature.^40^ This combination would likely be the best methodology to pursue when considering the feasibility, resource allocation, and statistical acceptability when designing future simulation OSCEs of resuscitation competence. Admittedly, only three of the five centres demonstrated this finding. Nevertheless, our analyses suggest the Toronto and Calgary centres contained very homogeneous groups of trainees. Recruitment at each of these centres was complicated by a non-uniform distribution of participating trainees’ abilities.

Our methodology and results were not without limitations. Although this study did not reproduce ICC and generalizability (*G*) values as strong as the previous single centre QSAT study,^32^ this was not surprising as there were many variables that were less easily controlled for (e.g., different simulation lab environments at test centres, variable trainee experience in simulation education and assessment, and non- standardized resident recruitment). As well, the feasibility of trialing more than 3 OSCE stations at 5 separate centres was too great due to limitations of time and resources at the various centres. As a result, the 3-station OSCE was not as generalizable as was hoped and future studies would require a more comprehensive approach with six or more stations to decrease trainee by scenario variance component. Nevertheless, our study provides the first multi-centre exploration of assessment of EM postgraduate trainees’ resuscitation skills in a dynamic, high fidelity simulation environment.

Currently, the Anaesthesia Training Program in Israel employs a high stakes simulation-based OSCE examination for its post-graduate trainees. With its centralized approach and testing centre, every trainee undergoes competency-based testing in a simulation environment to fulfill licensing requirements. Creating a similar system of assessment is consistent with the desired direction of the ACGME (USA), the FMEC (Canada), and the Ottawa conference proceedings (International).^4^–^6^

Looking forward, the next step will be to develop a competency-based summative assessment resuscitation OSCE with a centralized single centre model with multi-centre recruitment, with six or more scenarios, and one expert examiner per station. Such a study could focus on senior level residents alone and work towards an appropriate benchmarking strategy for competency thresholds for high stakes pass/fail summative examination. As well, a single centre model would help standardize trainee recruitment, OSCE station execution, and training of expert raters.

### Conclusion

In this study, we conducted a 3-station simulation-based OSCE at 5 academic EM training programs across Canada. We assessed the performance of EM postgraduate trainee resuscitation skills using the QSAT, a simple and modifiable competency-based assessment tool that has been validated previously at Queen’s University. Our study demonstrates a framework by which competency-based assessment can be performed in a simulation setting with a tool that shows promise in its discriminatory capabilities, inter-rater reliabilities, and generalizability. Future research should be directed at developing a multi-centre, single centre, multi-station OSCE format that further tests the use of this assessment system.

## Figures and Tables

**Figure 1:**
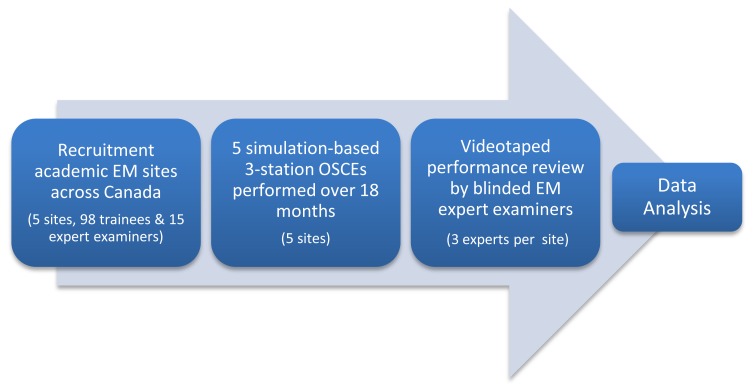
Study protocol flow chart

**Table 1:** OSCE enrolment

OSCE enrolment	Queen’s University	University of Toronto	University of Ottawa	University of Calgary	Dalhousie University	Total
PGY-1,2 (FRCP)	8	7	15	8	3	41
PGY-3 (CCFP-EM)	7	0	0	4	7	18
PGY-3,4,5 (FRCP)	10	10	10	3	6	39
Total	25	17	25	15	16	98

**Table 2:** Intraclass correlation coefficients for 3 scenarios across 5 sites (3 raters)

ICC	Queen’s University	University of Ottawa	University of Toronto	University of Calgary	Dalhousie University
Scenario 1	0.89	0.91	0.80	0.88	0.90
Scenario 2	0.88	0.89	0.65	0.97	0.86
Scenario 3	0.84	0.90	0.70	0.92	0.92

**Table 3:** Scenario score comparisons based on trainee level and program

		Scenario 1			Scenario 2			Scenario 3		
	n	Mean	SD	SE	Mean	SD	SE	Mean	SD	SE
PGY-1,2	41	14.27^a^	3.62	.56	14.41^a^	4.02	.63	13.79^a^	3.53	.55
CCFP-EM (PGY-3)	18	15.89	2.52	.59	15.02	2.89	.68	15.11	4.33	1.02
PGY-3,4,5	38	17.73	2.94	.48	17.79	2.81	.46	17.52	2.91	.47

Note:

a PGY-1,2 average scores are significantly lower than PGY-3,4,5 scores (*p*<0.001)

**Table 4:** Variance components and estimated generalizability coefficients for D –studies

Variance Component	Queen’s	Ottawa	Toronto	Calgary	Dalhousie
Trainee (σ^2^(*t*))	8.13	11.82	2.28	1.04	5.94
Rater (σ^2^(*j*))	2.40	1.10	0.55	0.04	0.50
Scenario (σ^2^(*c*))	0.70	1.15	0.00	2.16	0.00
Trainee X Rater (σ^2^(*tj*))	0.00	1.50	0.82	0.02	0.00
Trainee X Scenario (σ^2^(*tc*))	4.36	4.77	2.53	5.45	7.30
Rater X Scenario (σ^2^(*jc*))	0.27	0.20	0.65	0.04	0.60
Unexplained Var. (σ^2^(*tjc*))	2.79	2.79	3.99	1.37	3.48
σ^2^(δ) (1 Rater, 1 Scenario)	7.15	9.06	7.33	6.83	10.78
*Generalizability E(ρ^2^)*	0.53	0.57	0.24	0.13	0.36
*D-*Study Estimates					
*E(ρ^2^)* 1 Rater, 3 Scenarios	0.77	0.75	0.43	0.31	0.62
*E(ρ^2^)* 2 Raters, 3 Scenarios	0.81	0.81	0.54	0.34	0.66
*E(ρ^2^)* 3 Raters, 3 Scenarios	0.82	0.83	0.59	0.34	0.68
*E(ρ^2^)* 1 Rater, 6 Scenarios	0.87	0.81	0.55	0.47	0.77
*E(ρ^2^)* 2 Raters, 6 Scenarios	0.89	0.87	0.66	0.5	0.8
*E(ρ^2^)* 3 Raters, 6 Scenarios	0.9	0.89	0.71	0.51	0.81
*E(ρ^2^)* 1 Rater, 9 Scenarios	0.91	0.83	0.6	0.57	0.83
*E(ρ^2^)* 2 Raters, 9 Scenarios	0.93	0.89	0.71	0.6	0.86
*E(ρ^2^)* 3 Raters, 9 Scenarios	0.93	0.91	0.76	0.61	0.86

Note: σ^2^(δ) = σ^2^(*tj/n*′*_j_*) + σ^2^(*tc/n*′*_c_) +* σ^2^(*tjc/(n*′*_j_* *n*′*_c_)*)) Where *n*′*_j_* = number of raters *n*′*_c_* *=* number of scenarios*,* and *n*′*_j_* *n*′*_c_* = number of raters multiplied by the number of scenarios

*E(ρ^2^) =* σ^2^(*t*)/( σ^2^(*t*)+ σ^2^(δ))
